# MicroRNAs from plants to animals, do they define a new messenger for communication?

**DOI:** 10.1186/s12986-018-0305-8

**Published:** 2018-10-01

**Authors:** Zhiqing Li, Ruodan Xu, Ning Li

**Affiliations:** 1 State Key Laboratory of Medical Molecular Biology, Institute of Basic Medical Sciences, Chinese Academy of Medical Sciences, Peking Union Medical College, Tsinghua University, Beijing, 100005 People’s Republic of China; 2 Institute of Basic Theory for Chinese Medicine, China Academy of Chinese Medical Sciences, Beijing, 100700 People’s Republic of China; 3 Department of Engineering, Aarhus University, 8000 Aarhus, Denmark

**Keywords:** MicroRNAs (miRNAs), Small RNAs (sRNAs), Cross-kingdom communication, Gene regulation, Oral delivery, Herbal medicines, Hormones

## Abstract

MicroRNAs (miRNAs), a class of single-stranded non-coding RNA of about 22 nucleotides, are potent regulators of gene expression existing in both plants and animals. Recent studies showed that plant miRNAs could enter mammalian bloodstream *via* gastrointestinal tract, through which access a variety of tissues and cells of recipients to exert therapeutic effects. This intriguing phenomenon indicates that miRNAs of diet/plant origin may act as a new class of bioactive ingredients communicating with mammalian systems. In this review, in order to pinpoint the reason underlying discrepancies of miRNAs transmission from diet/plant to animals, the pathways that generate miRNAs and machineries involved in the functions of miRNAs in both kingdoms were outlined and compared. Then, the current controversies concerning cross-kingdom regulations and the potential mechanisms responsible for absorption and transfer of diet/plant-derived miRNAs were interpreted. Furthermore, the hormone-like action of miRNAs and the intricate interplay between miRNAs and hormones were implicated. Finally, how these findings may impact nutrition and medicine were briefly discussed.

## Background

MicroRNAs (miRNAs) are an extensive class of single-stranded non-coding RNA, 19–24 nucleotides in length, which negatively regulate gene expression at post-transcriptional level. Initiated by the discovery of *lin-4* in *Caenorhabditis elegans* (*C. elegans*) [1–3], thousands of miRNAs have been identified in plants, animals and other eukaryotes [4]. So far, over 38,589 miRNA gene loci distributed over 271 species have been stored in the latest miRBase (Release 22, March 2018, http://www.mirbase.org/). Through complementary base pairing, miRNAs can effectively inhibit translation or mediate degradation of target mRNAs (messenger RNAs). Both pleiotropically and ubiquitously, a single miRNA is often capable of recognizing hundreds of distinct mRNA transcripts. Likewise, one mRNA molecule usually has multiple miRNA binding sites and miRNAs binding to a single mRNA often act in a synergistic fashion [5]. MiRNAs play crucial roles in a wide range of biological processes, including developmental timing [6–8], cell differentiation [9–11], proliferation [12, 13], apoptosis [14–17] and metabolism [18], as such participate in a variety of human diseases [19–21]. It is estimated that 60% of all mammalian protein-coding genes are regulated by at least one miRNA [22, 23].

Our daily diet provides not only essential nutrients required for survival and growth but also bioactive compounds for health promotion and disease prevention [24]. Epidemiology studies have consistently shown that regular consumption of plant-based foods, such as fruit, vegetables and whole grains is beneficial for metabolic disease, cancer and age-related functional decline [25, 26]. However, the mechanistic understanding of the demonstrated impacts of plant-rich diets is still on their way to be clarified. It is generally believed that the bioactive components consumed from plants, such as flavonoids, phenolic acids and carotenoids contribute to lower risks of major chronic diseases [27]. Nonetheless, there is an intriguing phenomenon that synthetic supplements of phytochemicals are often not as efficient as complex plant materials, like vitamins, possibly implying the diversity of bioactive substances in plants and the existence of certain unidentified bioactive components.

In 2012, Zhang et al. [28] reported that diet/plant-derived miRNAs could be detected in the serum of human or plant-feeding animals, thus further regulate gene expression of recipients in a sequence-specific manner. Later on, Zhou [29] demonstrated that honeysuckle-encoded miRNA, miR2911, could be taken up *via* gastrointestinal (GI) tract of Influenza A virus (IAV)-infected mice and counteract viral infections. These findings constitute the initial clues that miRNAs may act as new bioactive constituents of plants and have the potential travelling from plants to animals *via* GI tract to access their cellular targets, influencing the physio-pathological conditions of their recipients. If the above described miRNAs transmission from plants to human (the so called cross-kingdom transmission) is validated, it may revolutionize our current knowledge of the properties, effectiveness and biological actions of bioactive compounds in diets [30, 31].

This review aims to summarize and analyze the main supporting and contradicting evidence of cross-kingdom regulation of miRNAs for a better comprehension of their potentials as a new effective constituent in plant-based diet and medication. To commence, the similarities and imparities of miRNAs between plants and animals are overviewed. For both kingdoms, the biogenesis of miRNAs, recognition of their targets and their mode of actions are particularly summarized and compared. Then, current evidence concerning cross-kingdom regulations of plant-derived miRNAs is outlined and the putative mechanisms of action responsible for efficient transfer from plant-derived sources to human cells are proposed. Furthermore, the hormone-like action of miRNAs and the intricate interplay between miRNAs and hormones are implicated. Finally, the potential impact of these findings on the progress of the study of nutrition and medicine is briefly discussed.

### Biogenesis of miRNAs

**Fig. 1:**
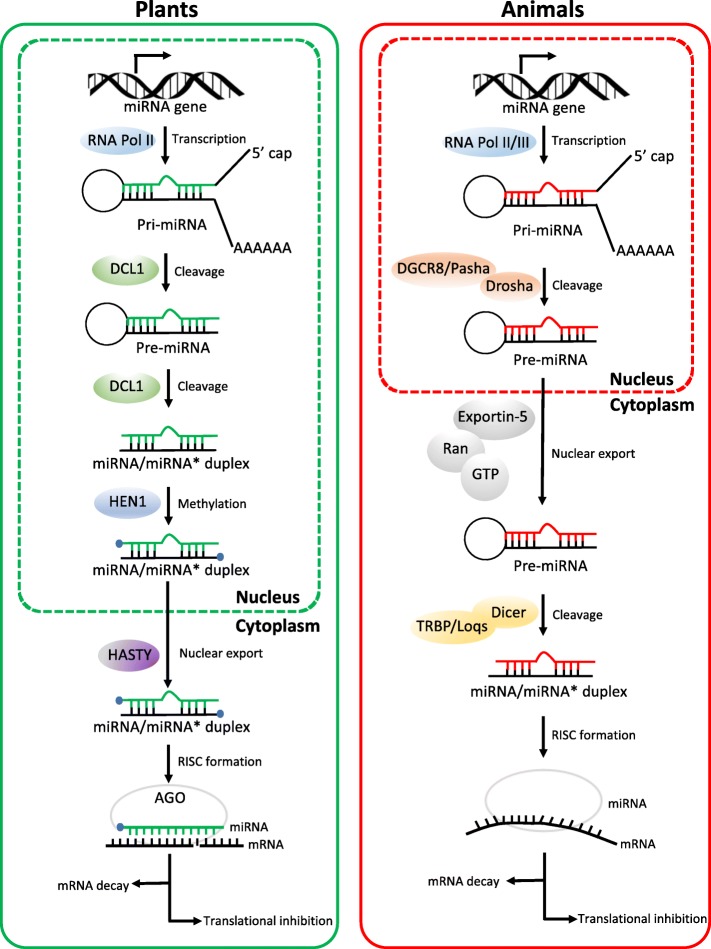
Comparison of miRNA biogenesis and activity pathways in plants and animals. Both in plants and animals, biogenesis of miRNAs initiates within the nucleus. In plants, miRNA/miRNA* duplexes are cleaved from pri-miRNAs through the action of DCL1 endonuclease in two steps. DCL1 firstly cuts off the imperfectly folded ends of pri-miRNAs to generate pre-miRNAs with stem-loop hairpin secondary structures. The resulting pre-miRNAs are further excised by DCL1 to mature miRNA/miRNA* duplexes. Then the 3′-terminal of duplexes is methylated by HEN1. By contrast, in animals, pre-miRNAs are produced in the nucleus by the action of the Drosha enzyme, together with its DGCR8 protein (in mammals) or Pasha protein (in flies). Duplexes of miRNA/miRNA* are further processed after exporting from nucleus to cytoplasm, where pre-miRNAs are cleaved by Dicer and TRBP (in mammals) or Loqs (in flies). In plants, HASTY is responsible for the transport of miRNA/miRNA* duplexes from nucleus to cytoplasm, whereas in animals, pre-miRNAs are recognized and then exported by Exportin-5 in a Ran-GTP-dependent manner. During RISC loading, one strand of the small RNA duplexes is selected as the guide strand (green in plants or red in animals) and incorporated into Ago to form a functional RISC, whereas the other strand is removed and degraded. In plants, miRNAs have near-perfect complementarity to their target mRNAs. By contrast, animal miRNAs often have targets with imperfect complementarity and the major determinant for animal miRNAs binding to their target mRNAs is a 6–8 nucleotide domain at the 5′ extremity or seed sequence. Arrows indicate the direction of the subsequent activity pathways. Both plant and animal miRNAs can regulate gene expression via mRNA decay and translational inhibition

Both in plants and animals, biogenesis of miRNAs initiates within the nucleus. Most miRNAs are transcribed by polymerase (Pol) II [32, 33], while a subset of animal miRNAs are products of RNA Pol III [34] (Fig. 1). The initial miRNA transcript is called primary miRNA (pri-miRNA), which contains a stable stem-loop structure. The pri-miRNA consists of thousands of nucleotides [35] and begins with a 7-methylguanosine (m^7^G) cap and ends with a 3′ poly(A) tail both in plants and animals. While, differences exist in the subsequent generating process of functionally mature miRNAs.

In animals, a long pri-miRNA is first cleaved by a ribonuclease (RNase) III, called Drosha, together with its double-stranded RNA (dsRNA)-binding protein partner DGCR8 (DiGeorge syndrome critical region gene 8, in mammals) or Pasha (in flies) [36–39], releasing a ∼ 70-nt hairpin precursor miRNA (pre-miRNA) within the nucleus. Afterwards, recognized by another RNase III Exportin-5, the pre-miRNA can be transported into the cytoplasm in a Ran (ras-related nuclear protein)-GTP (guanosine triphosphate)-dependent manner [40, 41]. Subsequently, another RNase III enzyme, Dicer, along with its dsRNA-binding partner transactivation-response RNA-binding protein (TRBP, in mammals) or Loquacious (Loqs, in flies) liberates pre-miRNA stem loop to generate a miRNA/miRNA* duplex [42–46]. In the cytoplasm, the miRNA/miRNA* duplex is separated; the miRNA strand is incorporated into RISC (RNA-induced silencing complex), whereas the miRNA* strand is typically degraded by an unknown mechanism. For slicing to occur, Argonaute (Ago), a core component of the RISC must be contained. Among the four mammalian Ago proteins, only Ago2 possesses “slicer” capabilities [47]. In cases in which cleavage is not possible, Ago proteins recruit additional protein partners to mediate silencing [48].

While in plants, due to the lack of Drosha genes, the cleavage of pri-miRNAs is carried out by the plant RNase-III-like protein. Among the four Dicer-like enzymes in plants, Dicer-like (DCL)1 is responsible for the bulk of biogenesis of miRNAs [49]. DCL1 mainly processes the pri-miRNA in two steps. It firstly cuts off the imperfectly folded end of the pri-miRNA to generate the pre-miRNA with a stem-loop hairpin secondary structure. The resulting pre-miRNA is further excised by DCL1 to produce a miRNA/miRNA* duplex [50]. Unlike animals, biogenesis of miRNA/miRNA* in plant is completed within the nucleus [51] in a nuclear processing center termed dicing bodies (D-bodies) [52, 53]. Another remarkable difference lies in that plant miRNAs are universally methylated at their 3′ ends while most products of animal miRNA genes are not. Following the releasing of the initial miRNA/miRNA* duplexes, they are then 3′-terminal 2′-O-methylated by a small RNA methyl transferase, called Hua Enhancer (HEN)1. This modification prevents their uridylation and subsequent degradation [54]. Methylated miRNA/ miRNA* duplexes are then exported from nucleus into cytoplasm for loading into cytoplasmic Ago proteins *via* a pathway controlled by the Hasty (HST), a plant homologue of Exportin-5 [51]. In comparison of animal miRNAs, ten Ago family members are present in plants, among which the predominant effector for miRNA-mediated reactions of silencing is Ago1 [55, 56] and most other plant Ago proteins are also likely to possess RNA cleavage activities [57].

### Target recognition of miRNAs

Identification of miRNA targets is the key for understanding miRNA function. The imperfect complementarity nature of base pairing between miRNAs and their mRNA targets in animals makes it challenging to systematically identify mRNA targets [22]. Functional recognition of miRNA target is known to principally comply with the seed rule, which defines that contiguous base pairing to the 5′ end of miRNAs, especially nucleotides 2–7, is crucial for targeting [58]. Such seed sites initially came from the first discovered miRNA *lin-4*, which had some sequence complementarity to multiple conserved sites within the *lin-14* RNA [1]. Hereafter, seed rule was further confirmed by large-scale studies monitoring whole transcriptome and proteome responses to miRNAs [59–63]. Practically, when applied in combination with evolutionary conservation [58], secondary structure [64] or neighboring context information [60] of mRNA, the seed rule is informatively valuable in prediction and analysis of canonical seed sites. However, there remain a certain number of target sites failed to be identified based on the perfect seed complementarity [65–68]. In fact, several biological studies have validated that perfectly matched miRNA seeds are neither necessary nor sufficient for detecting all functional miRNA-target interactions [69, 70]. Recent advances in a transcriptome-wide method of precisely mapping miRNA binding sites (Ago HITS-CLIP (High-throughput sequencing together with UV-crosslinking and immunoprecipitation) method) unexpectedly revealed that a large percentage of miRNA-mRNA interactions were not merely mediated by seed sites, but mediation by non-canonical sites required consideration [71, 72]. By far, the add-on modes of target recognition include pivot seed pairing or nucleation bulge [73], centering pairing miRNA-binding sites [74] and seed-like motifs [75–77].

In contrast to animal miRNAs, target recognition of plant miRNAs was primarily believed simple and straightforward, as most plant miRNAs had perfect or near-perfect complementarity to their target mRNAs. However, Brodersen et al. showed that translational repression was a widespread mechanism fulfilled by plant miRNAs [78], highlighting the enormous diversity and complexity of gene regulation in plant systems. Recent studies were more likely to reach a consensus on the ‘rules’ of base-pairing for a functional plant miRNA-target interaction: little tolerance of mismatches at positions 2–13, especially positions 9–11, and more tolerance of mismatches at 1 and 14–21 [79]. Although high complementarity between plant miRNA and their target was a prerequisite for silencing efficacy, deviations from this rule were also observed. One example was *Arabidopsis* miR398a, which post-transcriptionally regulated its target gene copper/zinc superoxide dismutase (CSD)2, despite the presence of a bulge and GU wobble in the crucial 2–13 region [80]. Similarly, another *Arabidopsis* miR398-blue copper-binding protein interaction had a 6-nt bulge between positions 6 and 7 of the miRNA [81]. Such studies indicated that complementarity might not be the sole determinant for efficient silencing; additional features, such as the accessibility or secondary structure of target mRNA [82], the RNA-binding proteins [83] and the stoichiometric ratio between miRNA and target mRNA abundance [84] should be taken into account.

With regard to target site positions in mRNAs, animal miRNAs were once thought to bind target mRNAs mainly on the 3′ untranslated region (UTR), while plant mRNAs that were regulated by miRNAs predominately located in the coding sequences (CDS). More insightful studies have provided evidence that both animal and plant miRNAs can target 5’ UTR, 3’ UTR and CDS [85, 86], suggesting the diversity of miRNAs binding sites and similarities in both kingdoms.

Successful target recognition of miRNAs relies additionally on their stabilities modulated by target transcripts. Previous studies have provided interesting evidence of reciprocal regulations between miRNAs and their target sequences, such that target RNAs can in turn modulate miRNA decay [87–89]. The extent of sequence complementarity appears to be crucial for miRNA degradation both in plants and animals [90]. In animals, as complementarity of miRNAs is limited to the seed for most endogenous targets and the unmethylated miRNA 3′ end may be leaved inside the Ago, miRNA degradation by target transcripts is uncommon [87]. However, exposure of a miRNA to an artificial target RNA with extensive pairing leads to miRNA 3′ trimming and tailing, perhaps *via* dislodging the miRNA 3′ end from Ago [87–89]. Compared with miRNAs in animals, plant miRNAs are nearly perfectly complementary to their target RNAs and extensive pairing may expose 3′ end of miRNAs [90]. When in the absence of methyltransferase activity of HEN1, an unmethylated miRNA is subject to 3′ trimming and tailing by enzymes [91]. Although the 2′-O-methylation on the 3′ terminal ribose markedly stabilizes plant miRNAs, methylated miRNAs can be degraded as well, which is possibly associated with demethylation [90]. Beyond target-dependent mechanisms, other factors impacting miRNA stability involve miRNA-degrading enzymes [92], cis-elements in miRNAs [93] and RNA-binding proteins [94]. To date, the dynamics of miRNA degradation and the associated molecular mechanisms remain largely obscure. Further effort will be needed to clarify how plant miRNAs are subject to degradation in animal kingdom system.

### Mechanism of miRNAs’ action

In both plants and animals, miRNAs need to form a ribonucleoprotein complex, RISC, to silence target mRNAs [95, 96]. The minimal RISC consists of a small RNA (sRNA) and Ago [97]. The miRNAs exert gene silencing through two main mechanisms: mRNA decay and translational repression [96]. The mode of target repression has been largely determined by the degree of complementarity between the miRNA and its mRNA targets. High complementarity, as seen in plants, promotes target cleavage by RISC, while seed-matching often leads to translational inhibition, which is believed the dominant mode of miRNAs’ action in animals [98]. In recent years, the boundaries between plant and animal miRNAs in terms of mechanisms of action are becoming blurry as there is more evidence showing that miRNA decay and translational inhibition can occur in both kingdoms. Even though, they may operate by mechanisms of fine distinctions.

#### miRNA-mediated mRNA decay in animals

Animal miRNAs were initially thought not to affect target mRNA levels, but only to repress translation [2, 99]. Subsequent studies in *C. elegans* and zebrafish showed that miRNAs also promoted the degradation of their target mRNAs [100], but the mechanism of mRNA decay was independent of endonucleolytic cleavage, which was different from that of plant miRNAs. In animals, there are normally three steps in miRNA-mediated mRNA decay. The first step is deadenylation. The miRNAs induce poly(A) shortening by recruiting two deadenylase complexes, carbon catabolite repressor (CCR)4-negative on TATA (NOT) and poly(A)-nuclease (PAN)2-PAN3, onto target mRNAs through GW182 (glycine-tryptophan protein of 182 kDa) proteins (trinucleotide repeat containing (TNRC)6 in mammals and GW182 or Gawky in *Drosophila*) [98, 101]. It is worth noting that GW182 proteins play a central role in animal miRNA pathway and function as flexible scaffolds to bridge the interaction between Ago proteins and downstream effector complexes, such as CCR4–NOT and PAN2–PAN3. In addition to deadenylases recruitment, GW182 proteins also promote the dissociation of poly(A)-binding protein (PABP), which could enhance the accessibility of the poly(A) tail to deadenylases, thereby increasing the efficiency of deadenylation [102]. The second step is decapping *via* the decapping protein (DCP)2, which requires additional cofactors for full activity or stability. These cofactors include DCP1, enhancer of decapping (EDC)3, EDC4 and DEAD box helicase 6 (DDX6; also known as Dhh1, RCK, p54 and Me31B in different species) [103]. Finally, deadenylated and decapped mRNAs are degraded by the major cytoplasmic nuclease 5′-to-3′ exoribonuclease (XRN)1 [98].

#### miRNA-mediated mRNA decay in plants

In contrast to animal miRNAs, plant miRNAs cannot promote deadenylation [104]; instead, they execute cleavage of target mRNA, which occurs at a precise position. This so called “slicing” is carried out by the P-element induced wimpy testes (PIWI) domain of Ago proteins, which forms an RNase H-like fold and exhibits endonuclease activity. The endonuclease activity of PIWI domain has been experimentally confirmed for *Arabidopsis* Ago1 [56, 105], as well as Ago2 [106], Ago4 [107], Ago7 [108] and Ago10 [109]. Upon slicing, two mRNA fragments are generated: one is 5′ fragment which is protected at its 5′ end by the cap structure with an unprotected 3′ end, and the other is 3′ fragment possessing an exposed 5′ end and a poly(A)-protected 3′ end. The two resulting fragments need to be cleared away swiftly, so that the RISC can move on to the next target. In *Arabidopsis,* XRN4, a 5′-to-3′ exoribonuclease, is responsible for degrading the 3′ fragments because accumulated abundance of 3′ fragments has been found in a loss-of-function mutant of XRN4 [110]. For the removal of the resulting 5′ fragments, there are different pathways that might contribute. Similar to 3′ fragments, the 5′ cleavage fragments can be degraded by XRN4. Additionally, the cytoplasmic exosome may also play a role, as its cofactor’s subunits, such as superkiller (SKI)2, SKI3 and SKI8, are required for the degradation of RISC-generated 5′ fragments [111]. Furthermore, the 3′ end of the 5′ fragment is frequently uridylated by HEN1 Suppressor (HESO)1 [112], which can accelerate the degradation of 5′ fragment.

#### miRNA-mediated translational repression in animals

Over the last few years, although considerable progress has been achieved in elucidating the biochemistry, biology and genomics of miRNA-directed mRNA regulation, the detailed mechanism of translational repression remains intensely debated. Broadly divided, translation includes three steps: ribosome initiation, elongation and termination. Interestingly, there is evidence for both initiation and post-initiation repression. With the advent of the ribosome profiling method, which allows accurate measurements of translation efficiencies, it is now accepted that inhibitory mechanisms occurring post-initiation could be ruled out [98]. The emerging consensus in this field is that miRNAs inhibit cap-dependent translation at initiation [113], but the precise molecular mechanism for this is ambiguous [114]. Translation initiation begins with eukaryotic translation initiation factor (eIF)4F complex binding to the cap structure of mRNA. EIF4F complex is comprised of the cap-binding protein eIF4E, the RNA helicase eIF4A and the scaffolding protein eIF4G, the latter of which interacts with both eIF4E and eIF4A. Proposed mechanisms for miRNA-mediated translational repression include GW182-mediated PABP displacement [102, 115], recruitment of the translational repressors through GW182 [116–119] and dissociation of eIF4A from eIF4F complex [120–122].

- GW182-mediated PABP displacement

GW182 proteins induce not only deadenylation and subsequent mRNA decay, but also translational repression [123]. PABP, which binds to the 3′ poly(A) tail of mRNAs, stimulates translation by stabilizing binding of eIF4F complex to the cap structure through interaction with eIF4G. Structural studies have revealed that GW182 proteins are capable of directly interacting with the C-terminal mademoiselle (MLLE) domain of PABP through PABP-interacting motif (PAM)2, therefore restraining PABP. Intriguingly, recent findings have demonstrated that miRNA promotes the dissociation of PABP from target mRNAs in a deadenylation-independent manner [102]. This phenomenon fits the proposed model positing that GW182-mediated displacement of PABP from the poly(A) tail breaks the ‘closed-loop’ structure formed by the interaction between eIF4G and PABP, thereby repressing translation initiation [124].

b. Recruitment of the Translational repressors through GW182

Through the CCR4-NOT complex, GW182 recruits downstream translational repressors, such as DDX6 or eIF4E transporter (4E-T) onto target mRNAs. DDX6 is known as a decapping activator but can also function in translational repression [125]. In mouse embryonic stem cells, DDX6 is shown to be recruited onto the miRNA targets through interacting with mammalian hyperplastic discs protein EDD (E3 ligase identified/isolated by differential display), which binds to GW182 proteins in the Argonaute-miRNA complexes [126]. More recent studies have also suggested that 4E-T, an eIF4E-binding protein, could be recruited onto the CCR4-NOT complex to suppress translation through DDX6, PATL (PAT1-like protein)1 or LSM (Sm-like domain-containing protein)14 [118, 119]. However, questions such as which step in translation is blocked by these repressors require further characterization.

c. miRNA-mediated dissociation of eIF4A

EIF4A RNA helicases are translation initiation factors that unwind secondary structures within 5’ UTR of mRNA, allowing the 43S pre-initiation complexes (PIC) to scan the 5’ UTR towards the start codon. Some recent studies have provided evidence that miRNAs could also repress translation *via* displacement of eIF4A from the cap-binding complex eIF4F, which blocks the 43S PIC recruitment or ribosomal scanning in vitro [120–122]. However, this model remains verification by direct evidence.

#### miRNA-mediated translational repression in plants

Plant miRNAs were initially supposed to silence target mRNAs only through endonucleolytic activity of Ago proteins. Strikingly, GW182 protein, the core component required for the miRNA-mediated translational repression in animals, is lacking in plants and its homolog proteins have not been found so far. However, the disproportional effects of miRNAs on target gene expression at the transcript versus protein level implied that plant miRNAs could induce translational repression in addition to target cleavage [127–129]. Early examples of miRNA-mediated translational inhibition in plants were Apetala (AP)2 and squamosa promoter binding protein-like (SPL)3 regulated by miR172 and miR156/7, respectively [127, 128]. When miR172 and miR156/7 accumulated abnormally, AP2 and SPL3 were altered at the protein levels compared with those of the wild-type, while their transcript levels were comparable. Similar observations were subsequently made for other miRNAs, including miR159 [130], miR164, miR165/6 [131], miR171, miR395, miR398 and miR834 [78]. Known factors involved in the translational repression comprise the microtubule-severing enzyme katanin1 (KTN1), the decapping activator Ge-1 homolog varicose (VCS) [78], the GW-repeat protein SUO [132], the ER membrane protein altered meristem program (AMP)1 and its homolog, like AMP (LAMP)1 [131]. Currently, it is far from clear how these factors exert their functions in the miRNA pathways of plant and how miRNA-mediated translational repression is regulated.

Taken together, the biogenesis and acting mechanism of miRNAs display a high degree of similarity between animals and plants even though there actually are several subtle differences.

### Diet/plant-derived miRNAs in animals

In fact, the natural uptake of diet-derived miRNAs or dsRNAs has been known to be biologically effective in a wide range of lower eukaryotes or invertebrates [31]. In 1998, it was first discovered that sRNAs found in dietary material could influence gene expression of ingesting organisms in *C. elegans* through the process of RNA interference [133]. Since then, it has been demonstrated that various invertebrate organisms could acquire sRNA molecules from diverse dietary sources by being orally exposed to dietary material containing either in vitro synthesized dsRNA [134, 135] or artificially expressed dsRNA of plant [136] or bacteria [137]. Continuing evidence has consistently shown that uptake of sRNA from environmental sources, including diet, is feasible in simpler metazoan organisms.

Interestingly, some recent evidence suggests that a similar phenomenon might occur in human and other mammals. The revolutionary report by Zhang et al. claimed that diet-derived plant miRNAs were found in the circulation and organs of human and mice, and they were sufficient and efficient to regulate human mRNAs [28]. Although it was earlier known that exogenous miRNAs could be detected in systemic fluids [138–141], this was the first evidence showing that a diet/plant-miRNA, namely rice-derived miR168a, was capable of regulating gene expression in its recipients. Notably, due to 2’-O-methylation on the terminal nucleotides, plant miRNAs are highly resistant to periodate oxidation. This characteristic structure of plant miRNAs renders authors feasibility and reliability to clearly distinguish plant-originated miRNAs from those of animals. In their report, rice-derived miRNAs were shown to target low-density lipoprotein receptor adapter protein (LDLRAP)1, a gene involved in cholesterol metabolism [28]. Functional studies in vitro and in vivo demonstrated that the binding of miR168a to LDLRAP1 mRNA inhibited LDLRAP1 expression in liver cells, and consequently decreased low-density lipoprotein (LDL) removal from the blood [28]. It is therefore proposed that intestinal epithelial cells could absorb plant miRNAs in food and package miRNAs into microvesicles (MVs) to shelter from degradation and subsequently release miRNAs into the circulation. The liberated miRNAs were then delivered *via* the blood stream to various tissues/cells where they modulated target gene expression [28]. In addition, they also implied that plant miRNAs could use even mammalian Ago2 protein to form their own RISC and perform their functions similar to those of mammalian miRNAs [28]. Though exciting, several groups have subsequently presented more intricate evidence arguing the possibility of plant miRNA uptake and their potential influences on biological processes in animals (Tables 1 and 2).

**Table 1:** Supporting evidence of cross-kingdom communication by diet/plant-derived miRNAs

Year	Contents	miRNAs involved	Source origin	miRNA levels	Potential mechanism	Function	Detection methods	Conclusion	Reference
2012	Plant miRNAs were present in human and animal sera and organs.	miR168a	Rice	fM Level	Associated with AGO2 complex and packaged in MVs	miR168a regulated mouse LDLRAP1 expression and consequently decreased LDL removal from mouse plasma.	HTS, RT-qPCR, Bioinformatics, NB, WB, AGO2 immunoprecipitation	Exogenous plant miRNAs in food could regulate the expression of target genes in mammals.	[28]
2014	miR172 from cabbage (*Brassica oleracea*) was detected in blood, spleen, liver and kidney of mice after feeding with plant extract.	miR172	Cabbage	Stomach contained about 4.5–0.4% (2–24 h after feeding), intestines 2.4–0.2% (2–36 h), blood 1.3–0.2% (2–72 h) and spleen 0.38–0.04% (2–72 h) of the miR172 orally administered.	sRNA could survive for more than 36 h in blood and fecal samples	No phenotypic changes were found in all the mice fed with the foreign RNA.	RT-qPCR, Electrophoresis	Exogenous plant miRNAs could survive in the murine GI tract, enter peripheral blood and continue to access other organs.	[142]
2015	Plant miRNAs were detectable in human plasma of volunteers after drinking juice.	18 plant miRNAs (miR156a, miR157a, miR158a, etc.)	Watermelon juice and mixed fruits	fM Level	largely encapsulated in MVs	Not mentioned	RT-qPCR, NB	Plant miRNAs in human plasma could be efficiently detected and reliably compared by RT-qPCR. Provided a SOP for measuring plant miRNAs in human and animal plasma.	[143]
2015	Even after an extensive pretreatment, plant-derived miRNA delivered by typical dietary ingestion remained bioavailable for uptake during early digestion.	miR166, miR167, miR168	Soybean and rice	*In vitro* methods	Not mentioned	Not mentioned	RT-qPCR	Storage, processing and cooking did not abolish plant miRNAs in food.	[149]
2014	miR2911 was highly stable in honeysuckle decoction, and continuous drinking or gavage feeding of honeysuckle decoction significantly elevated miR2911 levels in mouse blood and lung.	miR2911	Honeysuckle	fM Level	A unique sequence and high GC content, MVs-mediated pathway	miR2911 could directly target multiple viral genes and suppress viral infections.	HTS, RT-qPCR, NB, Fluorescent labeled tracing assay, Luciferase reporter assay, Ago2 immunoprecipitation	Provided evidence of physiological function of exogenous plant miRNAs in human and animals.	[29]
2015	Using a chow diet containing honeysuckle, plant-based sRNAs could be detected in sera and urine of mice	miR2911, miR168a	Honeysuckle	fM Level	Consumers of particular diets and/or with increased intestinal per- meability	Altered or damaged guts lining could enhance dietary miRNA uptake.	RT-qPCR, droplet digital PCR	Dietary sRNAs could survive circulation and are excreted in urine.	[144]
2015	miR2911 was detectable in sera and urine of the honeysuckle decoction-consuming mice.	miR2911	Dried herbs or flowers	fM Level	Circulating miR2911 was not bound by AGO2, but due to high GC content.	Not mentioned	RT-qPCR, AGO2 immunoprecipit-ation	The uptake of miR2911 might be a more commonplace phenomenon that could occur when eating a variety of plant-based foods.	[145]
2016	Plant-based miR2911 was measured 7 days after feeding in animals.	miR2911	Plants	fM Level	Circulating miR2911 was not associated with exosomes, but possibly with a protein.	Not mentioned	RT-qPCR	Mice consuming diets rich in vegetables displayed enhanced serum levels of plant specific miR2911.	[146]
2017	Plant-derived miR2911 was detectable in sera of mice fed with various vegetables.	miR2911	Cabbage *Arabidopsis*	miR2911 was detectable while other plant-based miRNAs failed to detect.	Increased levels of miR2911 correlated with the degradation of plant foods and rRNAs.	Not mentioned	RT-qPCR, Bioinformatic, Dual-luciferase reporter assay,	Provided insights into the atypical bioavailability of miR2911 and offered engineering strategies for plant-based sRNA therapeutics.	[147]
2015	Orally administered tumor suppressor miRNAs reduced tumor burden in Apc^Min/+^ mice and were detectable in intestinal tissue.	miR34a, miR143, miR145	Synthesized methylated miRNAs	Intestinal miR34a was at a detectable level; detection of miR143 and miR145 in mouse intestines were failed.	Not mentioned	Reduced tumor burden in the well-established Apc^Min/+^ mouse model of colon cancer.	RT-qPCR, Dissecting microscope.	Tumor suppressor miRNAs designed to mimic sRNAs produced in plants were taken up by the digestive tract of Apc^Min/+^ mice upon ingestion.	[150]
2016	Plant miR159 could be detected in human sera and tumor tissues, and was associated with breast cancer progression.	miR159	Synthesized methylated miRNAs	fM Level	Predominantly present in MVs	The miR159 in human serum was capable of inhibiting cell proliferation.	RT-qPCR, HTS, Dual-luciferase reporter assay, In situ hybridization, Immunohistochemistry, WB	The feasibility of using synthetic forms of plant miRNAs as dietary supplements in the treatment of human cancers, including those outside of the GI track.	[151]
2016	Strawberry fruit *Fv*miR168 affected properties of dendritic cells and their ability to respond to inflammatory stimuli.	*Fv*miR168	Strawberry fruit	biologically relevant amount	The immune-modulatory effect of plant miRNA was not sequence or plant specific.	Plant-based miRNAs modified dendritic cells ability to respond to inflammatory agents by limiting T cell proliferation.	RT-qPCR, Flow cytometry, Fluorescence microscopy	A potential for therapeutic use of plant miRNAs in the prevention of chronic inflammation related diseases.	[152]
2017	Ingestion of wild type blood increased the levels of miR451 and miR144 in peripheral blood of miR144/451-null mice	miR451 miR144	Wild type mice blood	At very low level but biologically relevant amount	Exosomes	Exogenous miR451 existing in miR144/451 knockout mice enhanced anti-oxidant activity *in vivo* via increasing the activity of Foxo3 pathway	Two different RT-qPCR, Dual-luciferase reporter assay, WB, FACS	miRNAs in foods or dietary supplements could affect the functions of the consumer.	[153]

*MVs* microvesicles, *HTS* high-throughput sequencing; *RT-qPCR* quantitative real time polymerase chain reaction, *NB* Northern blot, *WB* Western blot, *ELISA* enzyme-linked immunosorbent assay, *FACS* Fluorescent activated cell sorting, *SOP* standard operating procedure, *rRNAs* ribosomal RNAs, *fM* femtomole (10^−12^ mol/L)

**Table 2:** Contradicting evidence of cross-kingdom communication by diet/plant-derived miRNAs

Year	Contents	miRNAs involved	Source origin	miRNA levels	Refuting points	Detection methods	Conclusion	Reference
2013	Little or no plant miRNAs or miR168a were detected in blood or liver of mice fed with rice-containing diets.	miR168a	Rice	Unmeasurable	The observed changes in LDL levels might be due to the release of endogenous cholesterol stores in response to negligible dietary cholesterol intake in mice fed with only rice.	HTS, RT-qPCR, ELISA	Dietary exposure to miR168a did not affect plasma LDL levels. Plasma LDL changes reported by Zhang resulted from nutritional imbalances between test and control groups rather than an RNAi-mediated effect of consuming miR168a in rice.	[154]
2013	Plant miRNAs were not detectable in the plasma from healthy human subjects after intake of a western diet containing fruits.	miR156a miR159a miR169a	Plant material	Undetectable	Low measurable uptake	RT-qPCR	Horizontal delivery of miRNAs *via* oral ingestion of a typical diet was neither a frequent nor a prevalent event across multiple recipient animal organisms.	[158]
	Negligible expression of miR21 in plasma or organ tissue in miR21 knockout mice after oral diets replete with endogenous miR21.	miR21	Animal lard diet replete with miR21	Undetectable in plasma; less than one copy per cell in the liver, lungs, kidneys and stomach.				
	Negligible expression of miR156a, miR159a and miR169a in plasma or organs in mice after diets replete with these miRNAs.	miR156a miR159a miR169a	Vegetarian diets replete with these miRNAs	miR156a: far less than one copy of miRNA per cell in liver, lungs, kidneys and stomach; miR159a and miR169a: undetectable in either plasma and/or organs.				
	Negligible expression of plant-derived miRNAs in recipient honey bee tissues.	miR156a miR159a miR169a	plant-derived miRNA	Only miR156a but not miR159a or miR169a, was detected in abdominal tissue derived from nurses and foragers, but again at exceptionally low levels.				
2012	Predominant monocot miR168 sequence was present as a result of contamination from a non-plant source.	miR168a	Plant	Not available	Contamination	HTS, NB	The observed plant miRNAs in animal sRNA datasets could originate in the process of sequencing, and accumulation of plant miRNAs *via* dietary exposure was not universal in animals.	[155]
2014	Cross-contamination during library preparation was a source of exogenous RNAs.	miR168a miR156a miR167a	Plant	Not available	Contamination	HTS	Variable amounts of plant miRNAs were found in publicly available sRNA-seq data sets of human tissues.	[156]
2014	Failed to observe a postprandial increase in the brassica-specific miR824 or miR167a in broccoli sprouts feeding study.	miR167a miR824	Broccoli sprouts	Below detection limit	Low measurable uptake	RT-qPCR	Skeptical of the bioavailability and biologic activity of plant-borne miRNAs	[157]
2013	Nonhuman primates failed to uptake dietary plant miRNAs.	miR156 miR160 miR166 miR167 miR168 miR172	Fruit	Not available	The concentrations were too low to be specific and reliable.	RT-qPCR, droplet digital PCR	The level of miRNAs was too low to be true and/or amplification was non-specific.	[159]
2018	Corn miRNA was extensively degraded in the GI tract and that the uptake into circulation and tissues was minimal.	miR156a miR164a miR167a	Corn	No corn miRNAs could be detected in whole blood, fecal or liver of animals.	Significant degradation of corn miRNAs occurred during digestion.		No evidence of increased levels of corn miRNAs in whole blood or tissues after supplementation of corn miRNAs in the diet was observed in a mouse model.	[160]

*MVs* microvesicles, *HTS* high-throughput sequencing, *NB* Northern blot, *WB* Western blot, *ELISA* enzyme-linked immunosorbent assay, *LDL* low-density lipoprotein, *sRNA* small RNA

#### Supporting evidence (Table 1)

Following Zhang’s work, a study by Liang et al. showed that miR172 from cabbage (*Brassica oleracea*) was detected in the blood, spleen, liver and kidney of mice fed with plant total RNAs [142]. Moreover, they found that sRNAs could survive for 36 h or longer in blood and fecal samples in the presence of degrading enzymes, indicating the potential stability of sRNAs. In 2015, another group published their results obtained from volunteers drinking watermelon juice or eating mixed fruits (watermelon, banana, apple, orange, grape, mango and cantaloupe) [143]. Using real-time reverse transcription polymerase chain reaction (RT-qPCR) and northern blotting, they identified 10 plant miRNAs in human plasma at high basal levels [143]. By conducting a kinetics study, they proved that the absorption of plant miRNA was not a technical artifact or contamination, but a real physiological event. Importantly, they established a standard operation procedure for measuring plant miRNAs in human and animal plasma, which would promote investigations in this nascent field.

Studies supporting biologically relevant uptake of plant-originated miRNAs have focused on miR2911, a honeysuckle-derived miRNA [29, 144–147]. Honeysuckle is a well-known Chinese herb widely applied in the prevention and control of epidemic diseases. Modern pharmacological study has confirmed that honeysuckle has a broad spectrum of antimicrobial activity [148]. Zhang et al. [29] showed that continuous drinking or gavage feeding the decoction of boiled honeysuckle led to the elevation of miR2911 in the sera and lungs of mice. More interestingly, miR2911 was not degraded during the honeysuckle boiling process [29]. This phenomenon was consistent with a simulation study, in which dietary plant miRNAs were stably present in intact form after storage, processing, cooking and early digestion [149]. Subsequent functional studies of miR2911 demonstrated that miR2911 targeted and inhibited various IAVs, including H1N1, H5N1 and H7N9 [29]. They also demonstrated that the bulk of miR2911 in mouse peripheral blood was detected in MVs fraction and largely associated with the Ago2 complex, implying miR2911 might exert its function through the same MV-mediated pathway. Concerning the stability of miR2911, it was proposed to rely on its unique sequence and high GC content. More insightful knowledge of miR2911 was put forward by four independent studies from Yang et al. [144–147]. Consistently, Yang reported a significant increase of plant miR2911 in the sera and urine of the honeysuckle decoction-consuming mice [144]. Moreover, the damaged guts resulting from cisplatin also led to enhanced miRNA retention in the mouse circulation [144], which further verified the possibility of miRNAs transferring from plant to animals. Yang’ work [145, 146] also proposed that the high stability of circulating miR2911 was associated with neither exosomes nor Ago complex. Additionally, they showed that unlike most of plant-derived biomolecules, miR2911 was atypical as their abundance was positively correlated with the degradation of plant foods and rRNAs (ribosomal RNAs), and their biogenesis was Dicer independent [147]. The above information suggests that miRNAs may be one of the hidden but bioactive components in herbal medicines.

The potentials for therapeutic use of plant miRNAs were also supported by studies from other labs [150–153]. One group reported that the oral administration of a cocktail of three tumor suppressor miRNAs designed to mimic sRNAs produced in plants reduced tumor burden in a mouse model of colon cancer [150]. Another group showed that plant-derived miR159 could be predominantly detected in Western human sera and tumor tissues, and was associated with the incidence and progression of breast cancer [151]. More importantly, further data revealed that synthetic forms of plant miR159 significantly reduced breast tumor growth in mice by targeting sequence of the 3′ UTR of transcription factor (TCF)7 mRNA. Their study suggested that plant-engineered miRNA designed for specific targets possessed a significant potential in clinical therapeutic applications [151]. Supportively, Cavalieri et al. [152] demonstrated that strawberry miRNAs could act as ligands and bind to toll-like receptor (TLR)3 on dendritic cells, therefore modulating their responses to inflammatory agents. This study consolidated the therapeutic capacity of plant miRNAs in the prevention of human disorders. Using miR144/451 gene knockout mouse model, a more recent finding by Wang et al. [153] showed that exogenous miR451 were able to enter the circulation through digestive system of mouse to enhance anti-oxidant activity in vivo *via* Foxo3 pathway. These studies suggest that miRNAs derived from plants or diets can not merely transfer to animals effectively, but exert their gene-regulatory function in a cross-kingdom manner.

#### Contradicting evidence (Table 2)

Along with above mentioned studies supporting dietary uptake and biological function of plant-originated miRNAs, paramount efforts have been made arguing the rationality of this issue, including direct replications of Zhang’s work. Dickenson et al. [154] attempted to validate the original work of Zhang [28] but failed to find consistent evidence of dietary uptake of miR168a after rice feeding. In their study, mice were grouped into standard chow, a nutritionally sufficient diet containing 41% rice or raw rice, respectively. Unfortunately, little or no plant miRNAs or miR168a were detected in the blood or liver of mice fed with rice-containing diets. Interestingly, consistent with the result of Zhang [28], the levels of LDL in the mice liver were indeed increased in mice fed with uncooked rice, but the expression of LDLRAP1 remained unchanged across all three experimental groups. They therefore proposed that the increase in LDL levels reported by Zhang et al. was a result of short-time nutritional impact and possibly the release of endogenous cholesterol stores when cholesterol intake was insufficient in this experimental condition [154]. Using recently developed deep sequencing technologies and bioinformatic analysis, another two groups [155, 156] found variable amounts of plant miRNAs in public animal sRNA datasets. Among them, the most abundant molecule was miR168a, having a sequence typical of monocot plant species. Additionally, Zhang et al. [155] also conducted experiments with controlled insect feeding, in which plant-derived miRNAs including miR168a were also found in datasets for insects that did not feed with monocot plants. Based on experimental evidences proposing that cross-contamination during library preparation was an unnoticed source of exogenous miRNAs, Tosar et al. [156] directly pointed out that the underlying cause of findings achieved by Zhang and colleges was a contamination of miRNAs, instead of miRNAs transmitted from other kingdoms.

Besides, negative results regarding cross-species transmission of plant-derived miRNAs were obtained in various insects and animals [157–160]. Study by Baier et al. demonstrated that plant-borne miRNAs might be not bioavailable in human as the brassica-specific miR824 and plant-specific miR167a could be barely detectable in blood of four randomly selected participants after consumption of a broccoli sprout meal [157]. Consistently, Snow et al. found a substantial level of plant miR156a, miR159a and miR169a in a diet commonly consumed by human, mice and honeybees [158]. However, after ingestion of fruits full of listed plant miRNAs, all of investigated subjects did not carry detectable plasma levels of those molecules [158]. Similar negative findings were shown for *Macaca nemestrina,* a non-human primate model employed by Witwer et al. [159] when examining their responses to dietary intake of a miRNA-rich plant. The levels of certain plant miRNAs in blood were evaluated before and after (1, 4 and 12 h) ingestion by RT-qPCR and droplet digital PCR. Although they observed low concentrations of some investigated plant miRNAs, the amplification was variable and might be non-specific. Recently, another research group aimed to detect corn miRNAs in cecum, feces, liver and in whole blood of mice [160]. Similar to the studies discussed above, Huang and colleagues [160] failed to identify corn miRNAs in the mentioned organs following supplementation of corn miRNAs in animal diet or gavage to the animals. Further in vitro digestion system suggested that degradation of miRNAs was responsible for the observations [160]. Taken together, these independent investigations certified little or low measurable uptake of plant miRNAs in human and other mammals after consumption of plants or plant miRNAs and unfolded crucial problems existing in the study of cross-kingdom transmission of miRNAs.

The main stream of pitfalls claimed by dissenters is the reliability and sensitivity of techniques commonly applied in the study of cross-kingdom transmission of miRNAs. Firstly, contamination of endogenous miRNAs from recipients or study platforms could not be ruled out. Thus, a standard and consensus experimental protocol, which truly makes plant and animal miRNAs distinguishable, and accurate experimental performance in the absence of any known sources of plant contaminations are both highly recommended. Secondly, noises of background signals during RT-qPCR or artifacts from sequencing procedure raise great concerns, especially when low signals from target miRNAs were detected. This technical flaw necessitates reliable negative control groups and double-confirmation of positive results using independent methodologies. Thirdly, selection of relevant experimental controls gives rise to some discrepancies. A comprehensive design, application and analysis of more than one control group may assist to reduce the disagreement. Lastly, direct evidence of plant-originated miRNAs crossing GI may compromise the divergences existing in this field.

### A long travel: Delivery of diet/plant-derived miRNAs (Fig. 2)

Apart from controversies on whether diet/plant-derived miRNAs are sufficient and efficient to execute their regulation of gene expression in recipient cells in a cross-kingdom manner, concerns regarding how these molecules can survive the GI tract, enter circulatory system and ultimately recognize their targets have also been raised. The first crucial point is the stability of active diet/plant-derived miRNAs in the preparation process as well as in mammalian circulation. In the case of miRNAs from rice or herbal medicines, high-temperature processing such as cooking or boiling are unavoidable, in which miRNAs may be largely destroyed. In addition, the existence of RNases, phagocytosis and extreme pH in GI tract as well as blood circulation may also destabilize ingested miRNAs prior to their access to recipient cells. Fortunately, the presence of 2’-O-methylation on the 3′-terminal nucleotide seems to endow diet/plant-derived miRNAs with enhanced stability, ensuring their regulatory activity [50, 54]. Otherwise, the increased stability may also be explained by the unique sequence and GC content of plant miRNAs. The most prominent example is honeysuckle-derived miR2911 [29], as mentioned above. Beyond the innate capacity of diet/plant-derived miRNAs, the supposed existence of miRNA carriers is more likely to protect plant/diet-derived miRNAs in extremely harsh conditions and subsequently assist their translocation into mammals [30, 161]. According to their origin, size and mechanism of formulation, these carriers can be divided into: (1) exosomes (50–100 nm), which are derived from the endosomal membrane; (2) microvesicles (20 nm-1 μm), which are released from the plasma membrane during cell stress and (3) apoptotic bodies (1–5 μm), which are liberated in response to apoptotic stimuli [162, 163]. It has been reported that above listed vesicles could protect extracellular miRNAs against RNases on one hand, and on the other hand facilitate their transfer within the host [164, 165]. In 2014, Mu et al. [166] characterized edible plant derived exosome-like nanoparticles (EPDENs), in which proteins, lipids and miRNAs have been identified. Their data suggested that EPDENs were uptaken by intestinal macrophages and stem cells when orally administrated, and actively exerted biological functions on the recipient cells. This finding potentially implied EPDENs as possible mediators in the crosstalk between the plant kingdom and mammalian cells. Additionally, stabilization of extracellular miRNAs was proved to be associated with RNA-binding proteins, such as nucleophosmin 1 [167], high-density lipoproteins (HDL) [168] and Ago-2 [169].

**Fig. 2:**
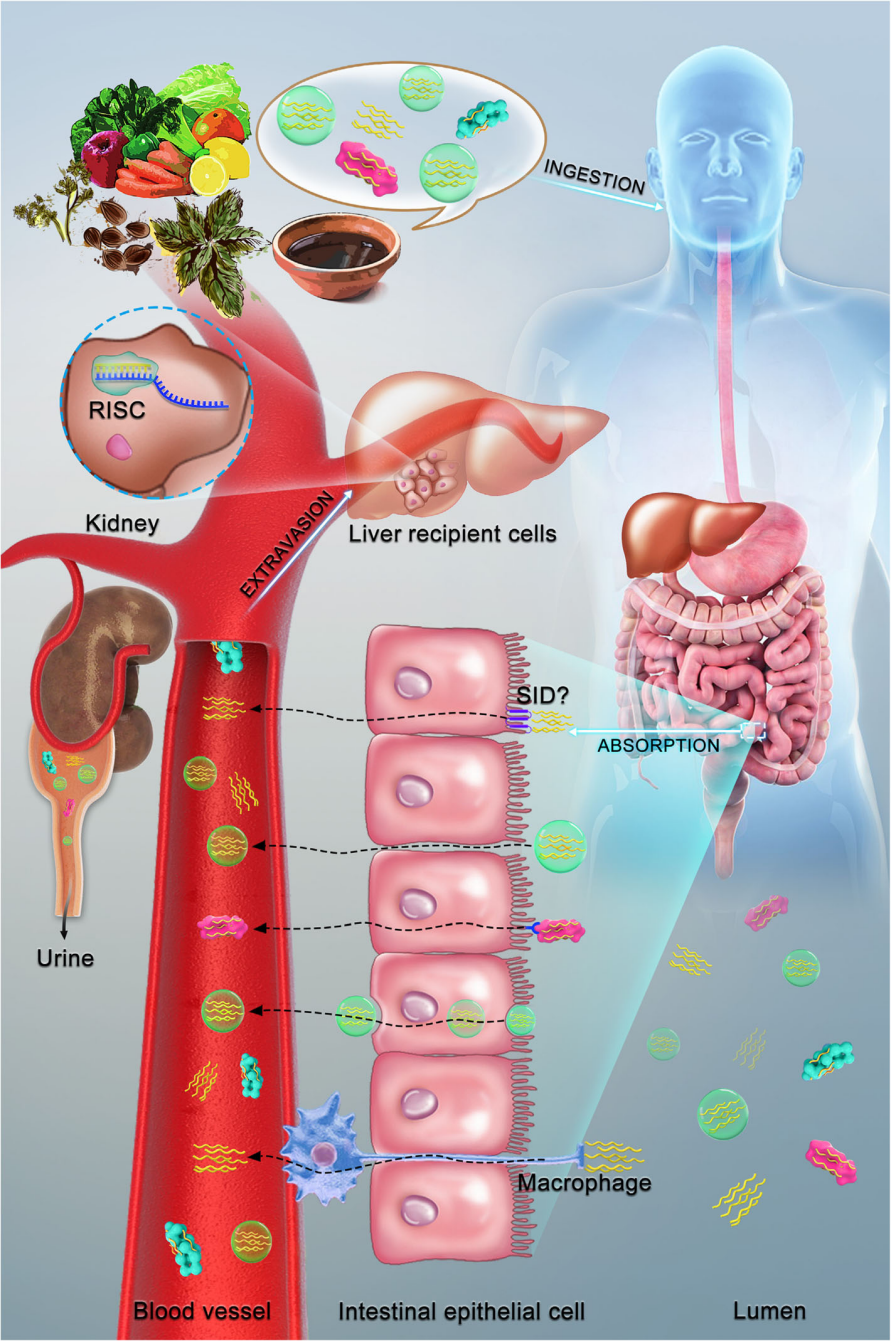
A schematic model of diet/plant-derived miRNAs journey from nature to human recipient cells. Plant-based foods or herb materials like in the form of decoctions are abundant in miRNAs, which are potentially packaged into vesicles or incorporated with proteins. When ingesting these plant materials, different forms of diet/plant-derived miRNAs could be released and subsequently transferred to the intestinal epithelial cells *via* different possible mechanisms, 1) by SID-like transporters; 2) vesicle-mediated transcytosis; 3) ribonucleoprotein complexes-mediated endocytosis; 4) through immune cells present in the gut barrier; 5) or diffusion in the space between epithelial cells. Being absorbed by gut lining, miRNAs enters circulation. Once delivered to targeted cells in organs such as liver and engulfed, diet/plant-derived miRNAs are liberated and subsequently execute their functionalities. If unfortunate, these circulating miRNAs may be filtrated and excreted at the kidney prior to their access to recipient cells

Another key issue is how diet/plant-derived miRNAs pass through the GI tract. By using an ex vivo everted gut sac to simulate the real physiological condition, a recent study by Luo et al. reported that exogenous plant miRNAs in food could cross the intestinal barrier and enter bloodstream of porcine, although the underlying mechanisms were unexplored [170]. In invertebrates, the transmembrane systemic RNA interference defective protein (SID)-1 allows sRNAs to be transported into cells outside of the digestive tract in *C. elegans* [171]. Meanwhile, SID-2 is another recently identified transmembrane protein in *C. elegans.* In contrast to the ubiquitous expression of SID-1, SID-2 is expressed in the intestine luminal membrane and might mediate the endocytosis of sRNAs from the lumen [172]. Notably, two homologous proteins of SID-1, namely SID-1 transmembrane family member (SIDT)1 and SIDT2 exist in most vertebrates, which may be involved in sRNA uptake in human [173, 174]. It is commonly believed that intestinal epithelial cells (IECs) form a continuous physical barrier in mammals, which provides a severe impediment against the uptake of environmental sRNAs [175]. Evidence to date defines two possible modes of transport across the digestive tract epithelium, either transcellular or paracellular. During the transcellular pathway, miRNAs could cross the intestine *via* transcytosis or protein transporters. Alternatively, some vesicles such as microvesicles or exosomes could also fuse with the epithelial cell membrane facilitating transportation. Additionally, for ribonucleoprotein complexes containing sRNAs, endocytosis has been shown to play a role in the uptake of sRNAs from dietary sources. A relevant example showed that miRNAs complexed with HDL can be endocytosed after interaction with HDL receptor scavenger receptor class B type I (SRBI) [168]. On the other hand, the paracellular pathway allows diffusion of molecules in the space between epithelial cells. This mode of transfer is usually under a delicate regulation of intercellular tight junctions [175]. Supportively, Yang et al. [144] proposed that intestinal injury or the microbiome within the GI tract could have a role by affecting the permeability of GI tract and further enhanced the delivery of dietary sRNAs into circulation. In addition to intestinal epithelial cells [175], the mammalian digestive tract is colonized by a variety of immune cells, which are able to trap sRNAs and other molecules on one side and release them on the other, with or without movement of the cell to a new location [176].

To sum up, the current model of miRNAs pathway from food sources to recipient cells may be built as follows: plant materials can be mechanistically crushed by oral activity and partially digested by various enzymes in our mouth/stomach. During these processes, plant miRNAs are released from destroyed cells and transferred to the intestinal epithelial cells, where plant miRNAs could be selectively incorporated with proteins or packaged into vesicles. Being absorbed by gut lining, miRNAs enters circulation. Once delivered to targeted cells and engulfed, plant miRNAs are liberated and subsequently execute their functionalities.

### miRNAs as novel hormone-like messengers

Diet/plant-derived miRNAs described above seem to act in a hormone-like fashion. Hormones are chemicals secreted from glands and enter the bloodstream where they circulate until exerting an effect on a downstream target cell. Both animal and plant cells use hormones for long-distance communication. Recent findings suggest circulating miRNAs as a novel form of cell-to-cell communicator [161, 177]. This viewpoint is reflected by the fact that circulating miRNAs are secreted by donor cells into circulation, then stably transported to other parts of the body and up-taken by recipient cells [178]. The reveal of hormone-like actions of miRNAs in recipient cells has driven this notion forward. Skog et al. showed that glioblastoma-specific proteins and RNAs (containing miR-21) were released in MVs and were converted by recipient normal cells into functional signals to stimulate proliferation and promote tumor progression [179]. In another study, miR-335 was indicated to modulate immune responses during the unidirectional transfer from T cells to antigen-presenting cells (APC) [180]. These results highly suggest that secreted miRNAs represent a novel mode of signaling for long-distance transportation of messages, by which donor cells can influence gene expression of recipient cells, and thus impact physiological and pathological processes.

With the discovery of circulating miRNAs in human body fluids, a more intricate level of cellular communication and regulation is introduced, and interactions between hormones and miRNAs are starting to emerge. Considering the significance of miRNAs and their involvement in various biological processes, it is not surprising that miRNAs are participating in the synthesis [181, 182] and secretion [183] of several hormones. Likewise, the expression of several miRNAs is in turn under regulations of hormones.

A finding has reported the role of miRNAs in adrenal cell physiology [184]. Angiotensin (Ang)II, the end product of the renin-angiotensin system, has been confirmed to up-regulate the expression of miR-21 in human adrenocortical cells (H295R) [184]. Notably, in microarray analysis of more than 200 miRNAs, only the expression level of miR-21 has been up-regulated by Ang II, and its overexpression caused an increase in aldosterone secretion and cell proliferation [184]. Another study showed that hormones could regulate miRNAs in the testis, ovary, and adrenal glands steroidogenic cells and the expression level of adrenal miRNAs appears to be regulated by more than one hormone [185]. Thyroid hormones (TH), which are known to be essential for the development, differentiation and maintenance of metabolic balance in mammals, might alter miRNA expression which could, in turn, alter mRNA abundance [186]. In Dong’s report, 40 significantly altered miRNAs has been detected in the livers of hypothyroid mice [186]. TH regulation of miRNAs was also supported by recent studies on miR-181d in hepatic cells [187]. Based on their data, two novel TH-regulated target genes that were downstream of miR-181d signaling, caudal type homeobox (CDX)2 and sterol O-acyltransferase (SOAT)2, have also been identified and characterized. Furthermore, the miR181d/CDX2/SOAT2 cascade was likely to contribute to this TH-dependent change in hepatic and systemic lipid metabolism [187]. In hypothalamus and pituitary, mounting miRNAs have been implicated as communicators interacting with hormones [188–190]. MiR-375 has been shown to inhibit the expression of proopiomelanocortin (POMC) and affect the synthesis and secretion of pituitary hormones [188]. Investigation of miR-24 has revealed its role in controlling the level of oxytocin, which was produced in the hypothalamic paraventricular and supraoptic nuclei [189]. Another study has demonstrated that miR-361-3p was involved in the secretory regulation of follicle-stimulating hormone (FSH) in porcine anterior pituitary cell [190].

Apart from crosstalk between hormones and miRNAs in mammals, their interactions appear to be rational in plants. The expression level of miR159 has been shown increased by abscisic acid (ABA), a plant hormone involved in bud and seed dormancy, root growth, leaf senescence and abscission, stomata opening and stress protection [191]. Besides, miR159, miR319 and miR166 have been proven to be involved in the plant hormone gibberellin pathway [192–194]. Hormone modulation of miRNAs in plant has been exemplified in the case of auxin. To keep normal development, change of exogenous auxin levels resulted in alterations of miR168 and miR169 levels [195, 196]. Another example of this scenario involves miR172 and miR319, which were discovered to be down-regulated when subjected to cytokinin 6-benzylaminopurine (6-BA) treatment [195]. Additionally, in the same report, Liu et al. has observed decreased levels of miR159 and miR394 when subjected to ethylene treatment in rice [195].

Together, miRNAs may act in a hormone-like fashion, and hormones and miRNAs are mutually regulated in both kingdoms. The interplay between the hormones and miRNAs may shed substantial light on whether hormones in human have any influence on diet/plant-derived miRNAs and how diet/plant-derived miRNAs coordinate with human hormones to fulfill their entire function.

## Conclusions and future perspectives

As potent intracellular mediators, functional significance of miRNAs has been widely validated. Evidence from animals showing inter-species gene regulation mediated by miRNAs derived from evolutionarily distant species suggests miRNAs as an alternative nutrient. Likewise, existence of miRNAs in herbal plants represents an extended scope of ingredients that may intentionally impact human health. In light of the phenomenon that synthetic supplements of phytochemicals do not usually have the same efficacy as complex herb materials, cross-species transmission of dietary miRNAs from plants to human may provide additional clues for evaluating the active therapeutic components of herbs.

At current stage, identification of more transmitted miRNAs in our diet and medical plants may greatly assist the enrichment and significance of food/herbal miRNA databases. However, the exact processes of plant-originated miRNAs crossing GI remain to be further validated. To fulfill the above, a consensus justifying the methodology of miRNA verification should be reached. Since mammals do not possess amplification pathways as *C. elegans*, quantification of biologically meaningful abundance of diet/plant-derived miRNAs in human body appears indispensable. In addition, mechanisms of how diet/plant-derived miRNAs are incorporated into the host RISC and further convey the silencing effects require delicate explanation. If these should be unrealistic, the exact mechanisms of intestinal absorption, bioavailability, tissue distribution and function of exogenous miRNAs could be envisaged, which though constitutes the major challenges will facilitate the development of both nutrition and medicine.

## Abbreviations

4E-T: EIF4E transporter
6-BA: 6-benzylaminopurine
ABA: Abscisic acid
Ago: Argonaute
AMP: Altered meristem program
Ang: Angiotensin
AP: Apetala
APC: Antigen-presenting cells
*C. elegans*: *Caenorhabditis elegans*
CCR: Carbon catabolite repressor
CDS: Coding sequence
CDX: Caudal type homeobox
CSD: Copper/zinc superoxide dismutase
DCL: Dicer-like
DCP: Decapping protein
DDX: DEAD box helicase
DGCR: DiGeorge syndrome critical region
dicing bodies: D-bodies
dsRNA: Double-stranded RNA
EDC: Enhancer of decapping
EDD: E3 ligase identified/isolated by differential display
eIF: Eukaryotic translation initiation factor
EPDENs: Edible plant derived exosome-like nanoparticles
FSH: Follicle-stimulating hormone
GI: Gastrointestinal
GTP: Guanosine triphosphate
GW182: Glycine-tryptophan protein of 182 kDa
Hasty: HST
HDL: High-density lipoproteins
HEN: Hua Enhancer
HESO: HEN1 Suppressor
HITS-CLIP: High-throughput sequencing together with UV-crosslinking and immunoprecipitation
IAV: Influenza A virus
IECs: Intestinal epithelial cells
KTN: Katanin
LAMP: Like AMP
LDL: Low-density lipoprotein
LDLRAP: Low-density lipoprotein receptor adapter protein
Loqs: Loquacious
LSM: Sm-like domain-containing protein
m7G: 7-methylguanosine
miRNA: MicroRNA
MLLE: Mademoiselle
mRNA: Messenger RNA
MV: Microvesicle
NOT: Negative on TATA
PABP: Poly(A)-binding protein
PAM: PABP-interacting motif
PAN: Poly(A)-nuclease
PATL: PAT1-like protein
PIC: Pre-initiation complexes
PIWI: P-element induced wimpy testes
Pol: Polymerase
POMC: Proopiomelanocortin
pre-miRNA: Precursor miRNA
pri-miRNA: Primary miRNA
Ran: Ras-related nuclear protein
RISC: RNA-induced silencing complex
RNase: Ribonuclease
rRNA: Ribosomal RNA
RT-qPCR: Quantitative real time polymerase chain reaction
SID: Systemic RNA interference defective protein
SIDT: SID1 transmembrane family member
SKI: Superkiller
SOAT: Sterol O-acyltransferase
SPL: Squamosa promoter binding protein-like
SRBI: Scavenger receptor class B type I
sRNA: Small RNA
TCF: Transcription factor
TH: Thyroid hormones
TLR: Toll-like receptor
TNRC: Trinucleotide repeat containing
TRBP: Transactivation-response RNA-binding protein
UTR: Untranslated region
VCS: Varicose
XRN: Exoribonuclease
