# Correction to: MicroRNAs from plants to animals, do they define a new messenger for communication?

**DOI:** 10.1186/s12986-018-0311-x

**Published:** 2018-10-18

**Authors:** Zhiqing Li, Ruodan Xu, Ning Li

**Affiliations:** 1 State Key Laboratory of Medical Molecular Biology, Institute of Basic Medical Sciences, Chinese Academy of Medical Sciences, Peking Union Medical College, Tsinghua University, Beijing, 100005 People’s Republic of China; 2 Institute of Basic Theory for Chinese Medicine, China Academy of Chinese Medical Sciences, Beijing, 100700 People’s Republic of China; 3 Department of Engineering, Aarhus University, 8000 Aarhus, Denmark

## Correction

Following publication of the original article [1], the author reported an error that was introduced during the production process. A part of Fig. 1 was missing in the article. The figure in the original article has now been corrected.

**Fig. 1:**
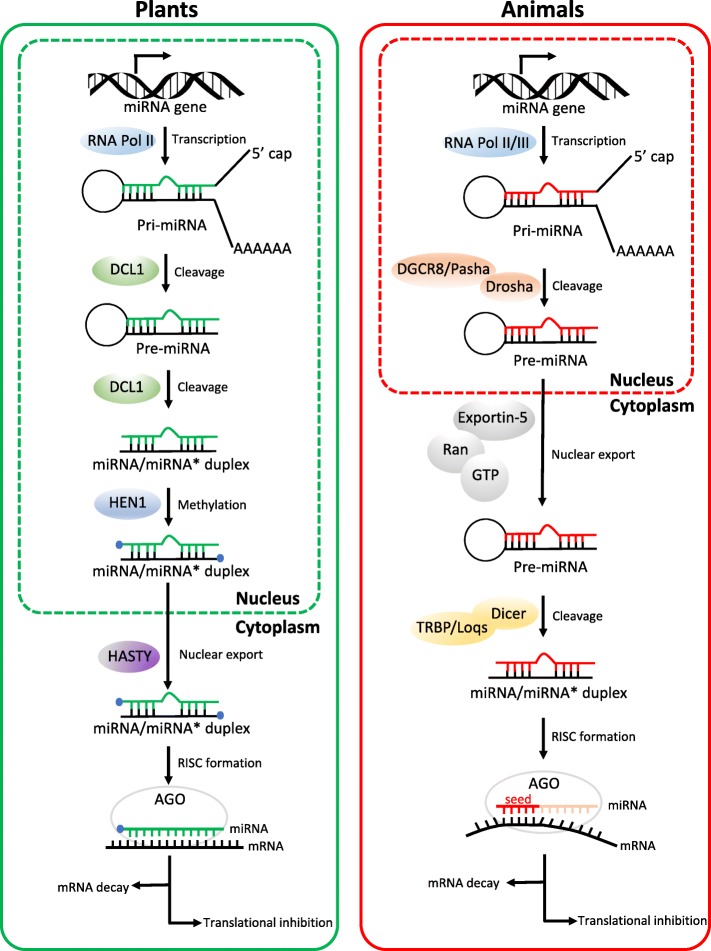
Comparison of miRNA biogenesis and activity pathways in plants and animals. Both in plants and animals, biogenesis of miRNAs initiates within the nucleus. In plants, miRNA/miRNA* duplexes are cleaved from pri-miRNAs through the action of DCL1 endonuclease in two steps. DCL1 firstly cuts off the imperfectly folded ends of pri-miRNAs to generate pre-miRNAs with stem-loop hairpin secondary structures. The resulting premiRNAs are further excised by DCL1 to mature miRNA/miRNA* duplexes. Then the 3′-terminal of duplexes is methylated by HEN1. By contrast, in animals, pre-miRNAs are produced in the nucleus by the action of the Drosha enzyme, together with its DGCR8 protein (in mammals) or Pasha protein (in flies). Duplexes of miRNA/miRNA* are further processed after exporting from nucleus to cytoplasm, where pre-miRNAs are cleaved by Dicer and TRBP (in mammals) or Loqs (in flies). In plants, HASTY is responsible for the transport of miRNA/miRNA* duplexes from nucleus to cytoplasm, whereas in animals, pre-miRNAs are recognized and then exported by Exportin-5 in a Ran-GTP-dependent manner. During RISC loading, one strand of the small RNA duplexes is selected as the guide strand (green in plants or red in animals) and incorporated into Ago to form a functional RISC, whereas the other strand is removed and degraded. In plants, miRNAs have near-perfect complementarity to their target mRNAs. By contrast, animal miRNAs often have targets with imperfect complementarity and the major determinant for animal miRNAs binding to their target mRNAs is a 6–8 nucleotide domain at the 5′ extremity or seed sequence. Arrows indicate the direction of the subsequent activity pathways. Both plant and animal miRNAs can regulate gene expression via mRNA decay and translational inhibition
